# (2,4-Di­chloro­benzyl­idene)[2-(1*H*-indol-3-yl)eth­yl]amine

**DOI:** 10.1107/S2414314623007800

**Published:** 2023-09-12

**Authors:** Suganya Murugan, Anaglit Catherine Paul, Themmila Khamrang, Savaridasan Jose Kavitha, Venkatachalam Rajakannan, Madhukar Hemamalini

**Affiliations:** a Department of Chemistry, Government Arts and Science College for Women, Kodaikanal, Tamil Nadu, India; b Department of Chemistry, Mother Teresa Women’s University, Kodaikanal, Tamil Nadu, India; c Assistant Professor, Department of Chemistry, DM College of Science, Dhanamanjuri University, Imphal, Manipur-795 001, India; d Department of Crystallography and Biophysics, University of Madras, Guindy Campus, Chennai-600 025, Tamil Nadu, India; University of Aberdeen, United Kingdom

**Keywords:** crystal structure, hydrogen bonding, C—H⋯π inter­actions

## Abstract

In the title compound, C_17_H_14_Cl_2_N_2_, the mol­ecule exists in an *E* configuration with respect to the C=N bond of the Schiff base fragment. The dihedral angle between the indole ring system and the benzene ring is 80.86 (12)°. In the crystal, mol­ecules are connected by N—H⋯N hydrogen bonds, generating a *C*(7) chain extending along the *a*-axis direction. No aromatic π–π stacking occurs but weak C—H⋯π inter­actions are observed.

## Abstract 2

The mol­ecules of the title compound are linked by N—H⋯N hydrogen bonds, generating a *C*(7) chain extending along the *a*-axis direction.

## Graphical abstract

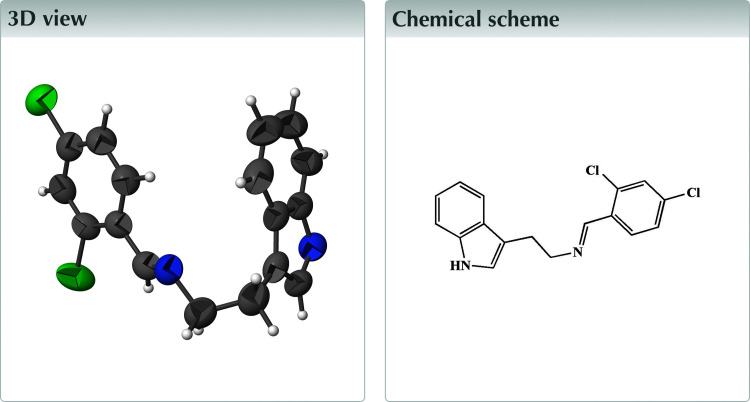

## Structure description

Schiff bases are widely used as catalysts, corrosion inhibitors and inter­mediates in organic synthesis, and also play a potential role in the development of coordination chemistry (Muralisankar *et al.*, 2016 ▸). Indole and its derivatives are useful staring compounds to derive pharmaceutical (Nalli *et al.*, 2020 ▸) and biological (Arumugam *et al.*, 2021 ▸) mat­erials. In the present study, the hydrogen-bonding inter­actions and C—H⋯π inter­actions of the title compound are investigated.

**Figure 1:**
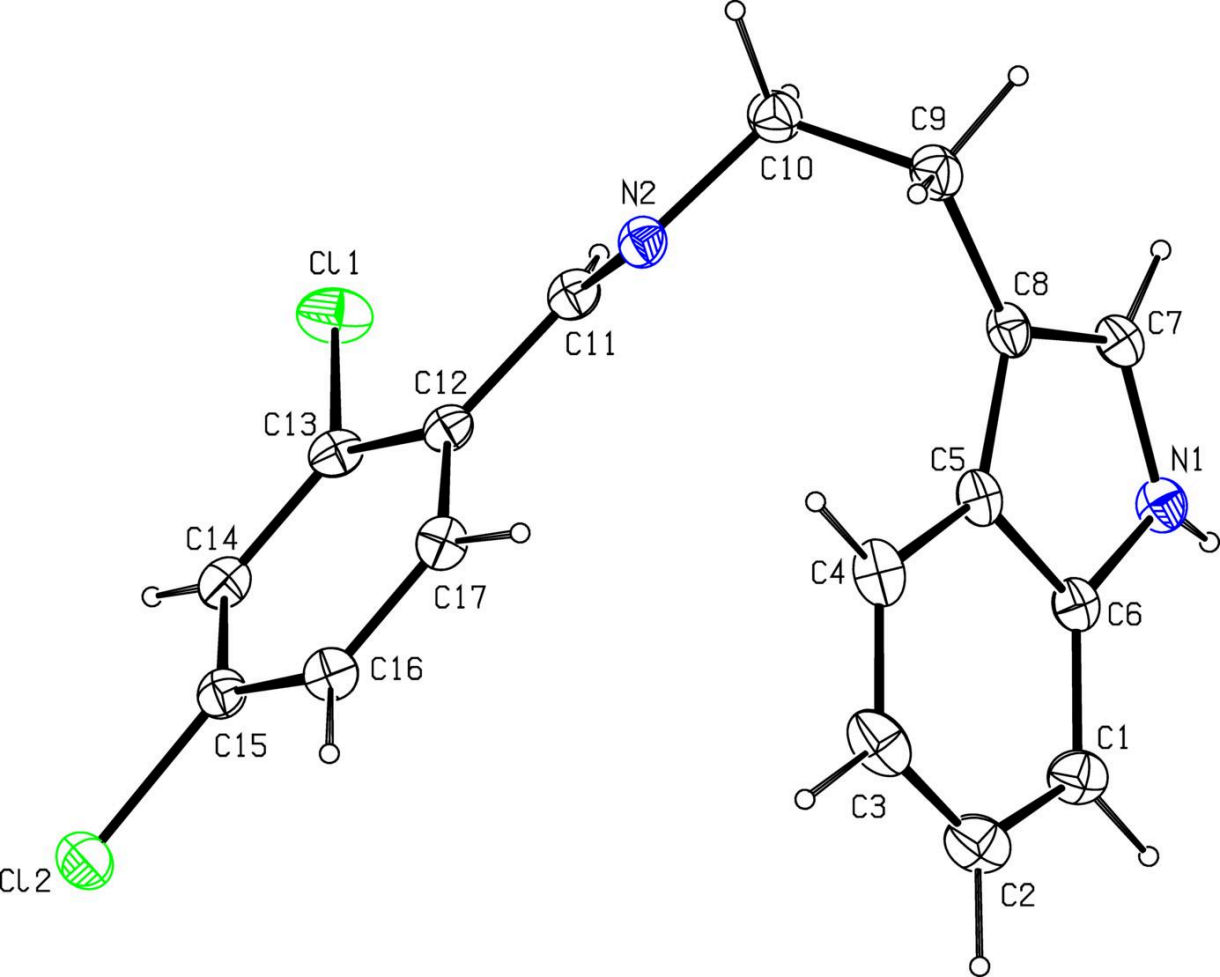
The mol­ecular structure of the title compound showing 50% displacement ellipsoids.

The asymmetric unit of the title compound is shown in Fig. 1 ▸. The C=N double bond adopts an *E* configuration. The bond lengths and angles in the title mol­ecule are normal and agree with those in other indole–imine compounds (*e.g.*, Suresh *et al.*, 2016 ▸; Ho *et al.*, 2006 ▸). The dihedral angle between the C1–C8/N1 indole ring system and the C12–C17 benzene ring is 80.86 (10)°.

**Figure 2:**
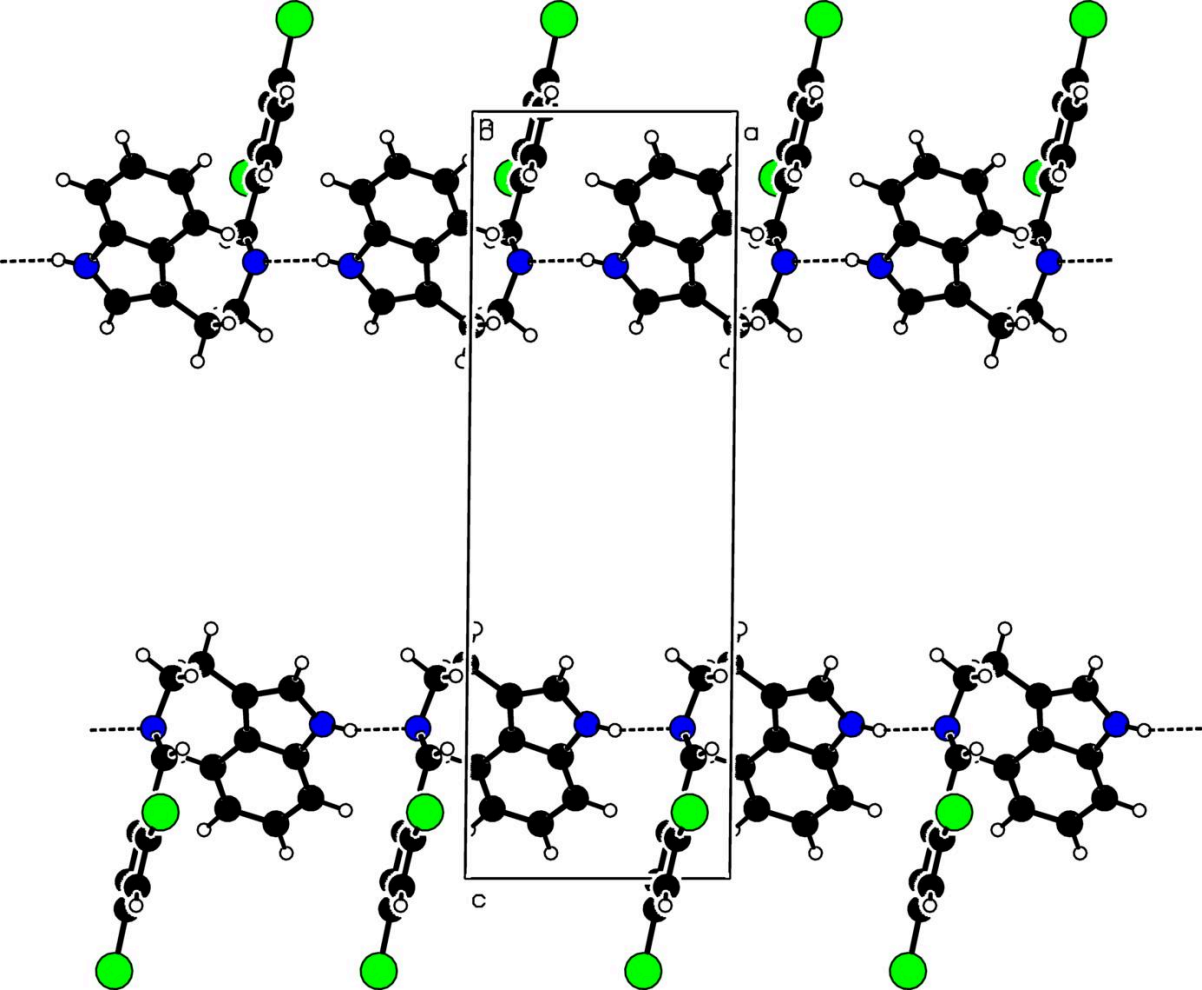
Partial packing diagram for the title compound showing the formation of [100] hydrogen-bonded chains.

In the extended structure, the N1—H5 group is a hydrogen-bond donor to atom N2 of the imino group (Table 1 ▸). These hydrogen bonds generate a *C*(7) chain extending along the *a*-axis direction, as shown in Fig. 2 ▸. There are no π–π inter­actions in this crystal structure but weak C—H⋯π inter­actions occur.

A search of the Cambridge Structural Database (Version 5.43, update November 2022; Groom *et al.*, 2016 ▸) for the benzyl­idene)-[2-(1*H*-indol-3-yl)-eth­yl]-amine skeleton yielded the hits 1-(anthracen-9-yl)-*N*-[2-(1*H*- indol-3-yl)eth­yl]methanimine (CSD refcode TEGJIB; Faizi *et al.*, 2017 ▸), 2-[2-(1*H*-indol-3-yl­ethyl­imino­meth­yl)]-5-methyl­phenol (PEVXEW; Brink *et al.*, 2018 ▸), *rac*-4-{(*E*)-[1-cyano-1-cyclo­hexyl-2-(1*H*-indol-yl)eth­yl]imino­meth­yl} benzo­nitrile (OCEWIE; Letessier *et al.*, 2011 ▸), 1*H*-indole-3-ethyl­enesalicylaldimine (FAJVIV; Rodriguez *et al.*, 1987 ▸) and 1-(4-chloro­phen­yl)-2-{[2-(1*H*-indol-3-yl) eth­yl]imino}-2-(4-meth­oxy­phen­yl)ethan-1-one (AZUYUS; Li *et al.*, 2021 ▸).

## Synthesis and crystallization

The title compound was synthesized by condensing tryptamine, 2-(1*H*-indol-3-yl)ethan-1-amine (0.01 mmol) and 2,4-di­chloro­benzaldehyde (0.01 mmol), which were taken separately, dissolved in 40 ml of ethanol, then mixed, and heated on a water bath for one h, then kept for crystallization. After a few days, colourless plate-shaped crystals were obtained.

## Refinement

Crystal data, data collection and structure refinement details are summarized in Table 2 ▸.

## Supplementary Material

Crystal structure: contains datablock(s) global, I. DOI: 10.1107/S2414314623007800/hb4446sup1.cif

Structure factors: contains datablock(s) I. DOI: 10.1107/S2414314623007800/hb4446Isup2.hkl

Supporting information file. DOI: 10.1107/S2414314623007800/hb4446Isup3.cml

CCDC reference: 2290063

Additional supporting information:  crystallographic information; 3D view; checkCIF report

## Figures and Tables

**Table 1:** Hydrogen-bond geometry (Å, °)

*D*—H⋯*A*	*D*—H	H⋯*A*	*D*⋯*A*	*D*—H⋯*A*
N1—H5⋯N2^i^	0.83 (3)	2.17 (3)	2.971 (3)	163 (2)

Symmetry code: (i) 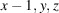 .

**Table 2:** Experimental details

Crystal data	
Chemical formula	C_17_H_14_Cl_2_N_2_
*M*_r_	317.20
Crystal system, space group	Monoclinic, *P*2_1_/*n*
Temperature (K)	296
*a*, *b*, *c* (Å)	7.2107 (8), 10.2179 (13), 20.863 (3)
β (°)	90.562 (4)
*V* (Å^3^)	1537.1 (3)
*Z*	4
Radiation type	Mo *K*α
μ (mm^−1^)	0.42
Crystal size (mm)	0.52 × 0.34 × 0.13

Data collection	
Diffractometer	Agilent Xcalibur, Atlas, Gemini
Absorption correction	Multi-scan (*SADABS*; Krause *et al.*, 2015 ▸)
*T*_min_, *T*_max_	0.631, 0.746
No. of measured, independent and observed [*I* > 2σ(*I*)] reflections	68672, 3872, 1946
*R*_int_	0.091
(sin θ/λ)_max_ (Å^−1^)	0.671

Refinement	
*R*[*F* ^2^ > 2σ(*F* ^2^)], *wR*(*F* ^2^), *S*	0.048, 0.134, 1.01
No. of reflections	3872
No. of parameters	246
H-atom treatment	All H-atom parameters refined
Δρ_max_, Δρ_min_ (e Å^−3^)	0.20, −0.27

Computer programs: *CrysAlis PRO* and *CrysAlis RED* (Agilent, 2012 ▸), *SHELXT2018/2* (Sheldrick, 2015*a* ▸), *SHELXL2018/3* (Sheldrick, 2015*b* ▸) and *PLATON* (Spek, 2020 ▸).

## full crystallographic data

### Crystal data

**Table:**

C_17_H_14_Cl_2_N_2_	*F*(000) = 656
*M**_r_* = 317.20	*D*_x_ = 1.371 Mg m^−^^3^
Monoclinic, *P*2_1_/*n*	Mo *K*α radiation, λ = 0.71073 Å
*a* = 7.2107 (8) Å	Cell parameters from 3778 reflections
*b* = 10.2179 (13) Å	θ = 2.6–29.9°
*c* = 20.863 (3) Å	µ = 0.42 mm^−^^1^
β = 90.562 (4)°	*T* = 296 K
*V* = 1537.1 (3) Å^3^	Plate, colourless
*Z* = 4	0.52 × 0.34 × 0.13 mm

### Data collection

**Table:**

Agilent Xcalibur, Atlas, Gemini diffractometer	1946 reflections with *I* > 2σ(*I*)
Radiation source: fine-focus sealed tube	*R*_int_ = 0.091
ω scans	θ_max_ = 28.5°, θ_min_ = 2.0°
Absorption correction: multi-scan (SADABS; Krause *et al.*, 2015)	*h* = −9→9
*T*_min_ = 0.631, *T*_max_ = 0.746	*k* = −13→13
68672 measured reflections	*l* = −27→27
3872 independent reflections	

### Refinement

**Table:**

Refinement on *F*^2^	Primary atom site location: dual
Least-squares matrix: full	Hydrogen site location: difference Fourier map
*R*[*F*^2^ > 2σ(*F*^2^)] = 0.048	All H-atom parameters refined
*wR*(*F*^2^) = 0.134	*w* = 1/[σ^2^(*F*_o_^2^) + (0.0498*P*)^2^ + 0.4436*P*] where *P* = (*F*_o_^2^ + 2*F*_c_^2^)/3
*S* = 1.01	(Δ/σ)_max_ = 0.001
3872 reflections	Δρ_max_ = 0.20 e Å^−^^3^
246 parameters	Δρ_min_ = −0.26 e Å^−^^3^
0 restraints	

### Special details

**Table:**

Geometry. All esds (except the esd in the dihedral angle between two l.s. planes) are estimated using the full covariance matrix. The cell esds are taken into account individually in the estimation of esds in distances, angles and torsion angles; correlations between esds in cell parameters are only used when they are defined by crystal symmetry. An approximate (isotropic) treatment of cell esds is used for estimating esds involving l.s. planes.
Refinement. All the H atoms were located in a difference Fourier map and allowed to refine freely (C—H = 0.93–0.96 and N—H = 0.83 Å).

### Fractional atomic coordinates and isotropic or equivalent isotropic displacement parameters (Å^2^)

**Table:**

	*x*	*y*	*z*	*U*_iso_*/*U*_eq_	
Cl2	0.82313 (10)	0.46440 (8)	0.37941 (3)	0.0896 (3)	
Cl1	0.65404 (13)	0.74406 (7)	0.58638 (4)	0.1034 (3)	
N2	0.6843 (2)	0.3930 (2)	0.69593 (9)	0.0656 (5)	
N1	0.0458 (3)	0.2537 (2)	0.70118 (10)	0.0701 (6)	
C5	0.3287 (3)	0.1772 (2)	0.68119 (11)	0.0628 (6)	
C8	0.3389 (3)	0.2540 (2)	0.73804 (11)	0.0648 (6)	
C6	0.1440 (3)	0.1792 (2)	0.65910 (11)	0.0631 (6)	
C12	0.6934 (3)	0.4814 (2)	0.58989 (12)	0.0593 (6)	
C7	0.1639 (3)	0.2993 (3)	0.74787 (13)	0.0680 (7)	
C13	0.6992 (3)	0.5929 (2)	0.55222 (12)	0.0641 (6)	
C14	0.7393 (3)	0.5892 (3)	0.48855 (14)	0.0683 (7)	
C15	0.7767 (3)	0.4709 (3)	0.46026 (12)	0.0648 (6)	
C17	0.7315 (3)	0.3640 (3)	0.55950 (14)	0.0675 (7)	
C11	0.6416 (3)	0.4833 (3)	0.65769 (13)	0.0661 (7)	
C16	0.7732 (3)	0.3571 (3)	0.49571 (14)	0.0707 (7)	
C4	0.4574 (4)	0.1057 (3)	0.64506 (16)	0.0818 (8)	
C10	0.6134 (4)	0.4015 (3)	0.76114 (14)	0.0785 (8)	
C1	0.0861 (5)	0.1121 (3)	0.60482 (14)	0.0836 (8)	
C3	0.4003 (6)	0.0415 (3)	0.59108 (17)	0.0979 (11)	
C9	0.5055 (4)	0.2801 (3)	0.77920 (14)	0.0796 (8)	
C2	0.2174 (6)	0.0439 (3)	0.57140 (17)	0.0972 (10)	
H12	0.720 (3)	0.289 (3)	0.5830 (12)	0.082 (8)*	
H11	0.573 (3)	0.556 (2)	0.6692 (10)	0.065 (7)*	
H10	0.543 (4)	0.476 (3)	0.7641 (12)	0.081 (9)*	
H9	0.726 (4)	0.407 (3)	0.7916 (13)	0.099 (9)*	
H8	0.586 (4)	0.201 (3)	0.7761 (12)	0.091 (9)*	
H4	0.579 (4)	0.105 (3)	0.6598 (12)	0.083 (9)*	
H13	0.797 (3)	0.275 (3)	0.4752 (12)	0.076 (8)*	
H14	0.743 (3)	0.663 (3)	0.4649 (12)	0.082 (8)*	
H7	0.470 (3)	0.290 (2)	0.8258 (12)	0.078 (7)*	
H6	0.126 (3)	0.357 (2)	0.7814 (10)	0.068 (7)*	
H1	−0.045 (4)	0.108 (3)	0.5888 (13)	0.106 (10)*	
H3	0.489 (4)	−0.005 (3)	0.5634 (15)	0.119 (11)*	
H2	0.177 (5)	−0.006 (4)	0.5331 (17)	0.132 (13)*	
H5	−0.060 (4)	0.282 (3)	0.6933 (12)	0.082 (9)*	

### Atomic displacement parameters (Å^2^)

**Table:**

	*U*^11^	*U*^22^	*U*^33^	*U*^12^	*U*^13^	*U*^23^
Cl2	0.0795 (5)	0.1146 (6)	0.0746 (5)	0.0090 (4)	0.0029 (3)	0.0035 (4)
Cl1	0.1558 (8)	0.0600 (4)	0.0939 (6)	0.0151 (4)	−0.0242 (5)	−0.0064 (4)
N2	0.0525 (11)	0.0758 (14)	0.0685 (13)	0.0038 (10)	−0.0010 (9)	0.0048 (11)
N1	0.0601 (13)	0.0747 (15)	0.0757 (15)	0.0114 (12)	0.0050 (11)	0.0011 (11)
C5	0.0652 (15)	0.0551 (14)	0.0681 (15)	0.0121 (11)	0.0125 (11)	0.0153 (12)
C8	0.0605 (14)	0.0739 (16)	0.0602 (14)	0.0068 (12)	0.0087 (11)	0.0181 (13)
C6	0.0695 (15)	0.0528 (14)	0.0671 (15)	0.0071 (12)	0.0078 (12)	0.0093 (12)
C12	0.0467 (12)	0.0621 (15)	0.0690 (15)	0.0059 (11)	−0.0094 (10)	−0.0004 (12)
C7	0.0707 (16)	0.0718 (17)	0.0617 (15)	0.0074 (13)	0.0119 (13)	0.0002 (13)
C13	0.0644 (14)	0.0555 (15)	0.0721 (16)	0.0053 (11)	−0.0151 (12)	0.0018 (13)
C14	0.0659 (16)	0.0587 (16)	0.0800 (19)	−0.0008 (12)	−0.0136 (13)	0.0104 (15)
C15	0.0478 (13)	0.0780 (18)	0.0684 (15)	0.0042 (12)	−0.0071 (10)	0.0065 (14)
C17	0.0659 (15)	0.0596 (16)	0.0769 (18)	0.0066 (12)	−0.0027 (12)	0.0089 (14)
C11	0.0534 (14)	0.0663 (17)	0.0783 (18)	0.0082 (12)	−0.0057 (12)	−0.0042 (14)
C16	0.0676 (16)	0.0640 (17)	0.0803 (19)	0.0123 (13)	−0.0014 (13)	−0.0037 (15)
C4	0.079 (2)	0.0709 (18)	0.096 (2)	0.0228 (15)	0.0201 (17)	0.0209 (17)
C10	0.0702 (18)	0.096 (2)	0.0690 (18)	0.0042 (17)	−0.0010 (14)	−0.0028 (16)
C1	0.102 (2)	0.0655 (17)	0.083 (2)	0.0028 (17)	−0.0044 (17)	−0.0025 (16)
C3	0.138 (3)	0.0652 (19)	0.091 (2)	0.031 (2)	0.032 (2)	−0.0007 (17)
C9	0.0741 (18)	0.098 (2)	0.0663 (18)	0.0031 (16)	−0.0018 (14)	0.0154 (16)
C2	0.137 (3)	0.0653 (19)	0.090 (2)	0.010 (2)	0.006 (2)	−0.0077 (17)

### Geometric parameters (Å, º)

**Table:**

Cl2—C15	1.724 (3)	C14—H14	0.90 (3)
Cl1—C13	1.733 (2)	C15—C16	1.378 (4)
N2—C11	1.256 (3)	C17—C16	1.369 (4)
N2—C10	1.461 (3)	C17—H12	0.92 (3)
N1—C6	1.365 (3)	C11—H11	0.92 (2)
N1—C7	1.369 (3)	C16—H13	0.95 (3)
N1—H5	0.83 (3)	C4—C3	1.364 (5)
C5—C6	1.405 (3)	C4—H4	0.93 (3)
C5—C4	1.406 (4)	C10—C9	1.514 (4)
C5—C8	1.424 (3)	C10—H10	0.92 (3)
C8—C7	1.362 (3)	C10—H9	1.03 (3)
C8—C9	1.494 (4)	C1—C2	1.371 (4)
C6—C1	1.385 (4)	C1—H1	1.00 (3)
C12—C13	1.385 (3)	C3—C2	1.377 (5)
C12—C17	1.386 (3)	C3—H3	0.99 (3)
C12—C11	1.467 (3)	C9—H8	1.00 (3)
C7—H6	0.96 (2)	C9—H7	1.01 (2)
C13—C14	1.363 (4)	C2—H2	0.99 (4)
C14—C15	1.373 (4)		
			
C11—N2—C10	117.5 (2)	N2—C11—C12	122.7 (2)
C6—N1—C7	108.9 (2)	N2—C11—H11	123.5 (14)
C6—N1—H5	123.4 (18)	C12—C11—H11	113.8 (14)
C7—N1—H5	125.7 (19)	C17—C16—C15	118.9 (3)
C6—C5—C4	117.4 (3)	C17—C16—H13	121.4 (15)
C6—C5—C8	107.8 (2)	C15—C16—H13	119.6 (15)
C4—C5—C8	134.7 (3)	C3—C4—C5	119.8 (3)
C7—C8—C5	105.8 (2)	C3—C4—H4	123.2 (17)
C7—C8—C9	126.5 (3)	C5—C4—H4	117.0 (17)
C5—C8—C9	127.7 (2)	N2—C10—C9	111.6 (3)
N1—C6—C1	130.4 (3)	N2—C10—H10	108.1 (16)
N1—C6—C5	107.1 (2)	C9—C10—H10	112.3 (17)
C1—C6—C5	122.5 (2)	N2—C10—H9	107.3 (15)
C13—C12—C17	116.5 (2)	C9—C10—H9	107.4 (16)
C13—C12—C11	123.1 (2)	H10—C10—H9	110 (2)
C17—C12—C11	120.4 (2)	C2—C1—C6	117.6 (3)
C8—C7—N1	110.4 (2)	C2—C1—H1	117.6 (17)
C8—C7—H6	126.3 (14)	C6—C1—H1	124.7 (17)
N1—C7—H6	123.3 (13)	C4—C3—C2	121.2 (3)
C14—C13—C12	122.5 (2)	C4—C3—H3	121.5 (19)
C14—C13—Cl1	117.9 (2)	C2—C3—H3	117.2 (19)
C12—C13—Cl1	119.5 (2)	C8—C9—C10	114.6 (2)
C13—C14—C15	119.2 (3)	C8—C9—H8	106.5 (16)
C13—C14—H14	121.4 (17)	C10—C9—H8	110.3 (15)
C15—C14—H14	119.4 (17)	C8—C9—H7	111.0 (13)
C14—C15—C16	120.5 (3)	C10—C9—H7	107.0 (14)
C14—C15—Cl2	119.7 (2)	H8—C9—H7	107 (2)
C16—C15—Cl2	119.8 (2)	C1—C2—C3	121.4 (3)
C16—C17—C12	122.4 (3)	C1—C2—H2	118 (2)
C16—C17—H12	120.0 (17)	C3—C2—H2	121 (2)
C12—C17—H12	117.5 (16)		
			
C6—C5—C8—C7	−0.3 (3)	C13—C14—C15—Cl2	178.70 (17)
C4—C5—C8—C7	179.1 (3)	C13—C12—C17—C16	0.2 (3)
C6—C5—C8—C9	179.4 (2)	C11—C12—C17—C16	177.4 (2)
C4—C5—C8—C9	−1.1 (4)	C10—N2—C11—C12	−175.7 (2)
C7—N1—C6—C1	179.5 (3)	C13—C12—C11—N2	−159.7 (2)
C7—N1—C6—C5	0.9 (3)	C17—C12—C11—N2	23.3 (4)
C4—C5—C6—N1	−179.9 (2)	C12—C17—C16—C15	−0.3 (4)
C8—C5—C6—N1	−0.4 (3)	C14—C15—C16—C17	0.1 (4)
C4—C5—C6—C1	1.4 (4)	Cl2—C15—C16—C17	−178.35 (18)
C8—C5—C6—C1	−179.1 (2)	C6—C5—C4—C3	−0.3 (4)
C5—C8—C7—N1	0.9 (3)	C8—C5—C4—C3	−179.7 (3)
C9—C8—C7—N1	−178.9 (2)	C11—N2—C10—C9	124.4 (3)
C6—N1—C7—C8	−1.1 (3)	N1—C6—C1—C2	−179.7 (3)
C17—C12—C13—C14	0.2 (3)	C5—C6—C1—C2	−1.4 (4)
C11—C12—C13—C14	−176.9 (2)	C5—C4—C3—C2	−0.7 (5)
C17—C12—C13—Cl1	−179.82 (17)	C7—C8—C9—C10	−89.3 (3)
C11—C12—C13—Cl1	3.1 (3)	C5—C8—C9—C10	91.0 (3)
C12—C13—C14—C15	−0.4 (4)	N2—C10—C9—C8	−60.8 (4)
Cl1—C13—C14—C15	179.62 (17)	C6—C1—C2—C3	0.3 (5)
C13—C14—C15—C16	0.2 (4)	C4—C3—C2—C1	0.8 (5)

### Hydrogen-bond geometry (Å, º)

**Table:**

*D*—H···*A*	*D*—H	H···*A*	*D*···*A*	*D*—H···*A*
N1—H5···N2^i^	0.83 (3)	2.17 (3)	2.971 (3)	163 (2)

Symmetry code: (i) *x*−1, *y*, *z*.
